# Pollen-vegetation richness and diversity relationships in the tropics

**DOI:** 10.1007/s00334-017-0642-y

**Published:** 2017-10-09

**Authors:** William D. Gosling, Adele C. M. Julier, Stephen Adu-Bredu, Gloria D. Djagbletey, Wesley T. Fraser, Phillip E. Jardine, Barry H. Lomax, Yadvinder Malhi, Emmanuel A. Manu, Francis E. Mayle, Sam Moore

**Affiliations:** 1 Institute for Biodiversity and Ecosystem Dynamics, University of Amsterdam, Amsterdam, The Netherlands; 2 Geography, Department of Social Sciences, Oxford Brookes University, Oxford, UK; 3 CSIR-Forestry Research Institute of Ghana, Kumasi, Ghana; 4 School of Environment, Earth and Ecosystem Sciences, The Open University, Milton Keynes, UK; 5 Institute of Earth and Environmental Sciences, Universität Potsdam, Potsdam, Germany; 6 Agricultural and Environmental Sciences, The University of Nottingham, Nottingham, UK; 7 Environmental Change Institute, School of Geography and the Environment, University of Oxford, Oxford, UK; 8 Department of Geography and Environmental Science, University of Reading, Reading, UK

**Keywords:** Neotropics, Palaeotropics, Palynology, Pollen trap, Forest-savannah, Savanna

## Abstract

Tracking changes in biodiversity through time requires an understanding of the relationship between modern diversity and how this diversity is preserved in the fossil record. Fossil pollen is one way in which past vegetation diversity can be reconstructed. However, there is limited understanding of modern pollen-vegetation diversity relationships from biodiverse tropical ecosystems. Here, pollen (palynological) richness and diversity (Hill *N*_1_) are compared with vegetation richness and diversity from forest and savannah ecosystems in the New World and Old World tropics (Neotropics and Palaeotropics). Modern pollen data were obtained from artificial pollen traps deployed in 1-ha vegetation study plots from which vegetation inventories had been completed in Bolivia and Ghana. Pollen counts were obtained from 15 to 22 traps per plot, and aggregated pollen sums for each plot were > 2,500. The palynological richness/diversity values from the Neotropics were moist evergreen forest = 86/6.8, semi-deciduous dry forest = 111/21.9, wooded savannah = 138/31.5, and from the Palaeotropics wet evergreen forest = 144/28.3, semi-deciduous moist forest = 104/4.4, forest-savannah transition = 121/14.1; the corresponding vegetation richness/diversity was 100/36.7, 80/38.7 and 71/39.4 (Neotropics), and 101/54.8, 87/45.5 and 71/34.5 (Palaeotropics). No consistent relationship was found between palynological richness/diversity, and plot vegetation richness/diversity, due to the differential influence of other factors such as landscape diversity, pollination strategy, and pollen source area. Palynological richness exceeded vegetation richness, while pollen diversity was lower than vegetation diversity. The relatively high global diversity of tropical vegetation was found to be reflected in the pollen rain.

## Introduction

Biodiversity is widely regarded as important for ecosystem function and services (Hooper et al. 2005; Cardinale et al. 2012), and consequently the observed loss of biodiversity over recent decades (Benitez-Malvido and Martinez-Ramos 2003; Stuart et al. 2004; Dirzo et al. 2014) has resulted in many efforts being made to preserve biodiversity. For example, actions have targeted the preservation of individual species (Teich et al. 2005), the establishment of areas of conservation priority (Myers et al. 2000) and the promotion of re-wilding at a continental scale (Navarro and Pereira 2012). The current observed loss of biodiversity is not unique in geological history with five other broad scale extinction events identified in the fossil record during the: (1) Late Ordovician, (2) Late Devonian, (3) Late Permian, (4) Late Triassic and (5) end-Cretaceous (Benton 1995; McGhee et al. 2004; Twitchett 2006).

The highest biodiversity for most organisms on Earth is concentrated in the tropics and, in part due to this, there has been a long-standing interest in past ecological change in the tropics. The first investigations into past ecological change from tropical regions came from Hawaii (Selling 1948), the Andes (van der Hammen and Gonzalez 1960), southern Africa (van Zinderen Bakker and Clark 1962), east Africa (Coetzee 1964), the Galapagos Islands (Colinvaux 1972), the Amazon basin (van der Hammen 1972) and west Africa (Maley and Livingstone 1983). The focus of early studies of fossil pollen from the tropics was on the nature of the past vegetation and the inferences that could be drawn about past climate conditions. However, as the number of fossil pollen records from tropical regions grew, these data were used to debate hypotheses on the origins of high tropical biodiversity (e.g. Colinvaux 1987; van der; Hammen and Hooghiemstra 2000; Bush et al. 2011). Despite c. 50 years of work in the tropics, large regions, particularly in lowland areas, remain relatively unexplored from a palaeoecological perspective (Flantua et al. 2015), mainly due to logistical and geographical constraints.

Variations in past diversity in the tropics have been inferred from changes in fossil bones, Mollusca and pollen (Hoorn et al. 2010). Based on the fossil pollen record, it is thought that vegetative diversity in the Neotropics has declined from a peak around 45 million years ago (Jaramillo et al. 2006). To be confident that observations of reduced biodiversity in the fossil record are an accurate reflection of biodiversity loss in the actual environment, it is important to understand the relationship between modern biodiversity and the way in which it is likely to be preserved in the fossil record.

The link between pollen and vegetation diversity is known to be complex due to the different taxonomic resolution possible, the variation in pollen production and dispersal between plants, and the accuracy with which the pollen sum captures the diversity of the sample (Goring et al. 2013; Birks et al. 2016). Furthermore, the relationship between pollen (palynological) diversity and its parent vegetation from the most biodiverse region of the world, the tropics, remains under-studied. Previous investigations of pollen diversity in the tropics have focused on the pollen morphological diversity within particular taxonomic groups (e.g. Caccavari 2002; Arce and Banks 2001), while diversity in pollen rain has been shown to follow trends in vegetation diversity over elevation gradients in the Andes (Weng et al. 2006; Jantz et al. 2013). Here we present the first systematic comparison of modern vegetation diversity, and the diversity of the pollen rain it produces from two regions of forest and savannah vegetation, one located in the New World (Neotropics) and the other in the Old World tropics (Palaeotropics).

## Methods

Modern pollen rain was collected in artificial pollen traps, following Gosling et al. (2003), from forest and savannah vegetation in Bolivia (Neotropics) and Ghana (Palaeotropics) (Table 1). All pollen traps were deployed for 1 year and were located in vegetation study plots of 1 ha (Gosling et al. 2009; Julier et al. 2017; Julier 2017) from which quantitative inventories of all plants > 10 cm diameter at breast height (dbh) had been obtained (forest plots), or all vegetation cover was recorded (savannah plots) (Panfil 2001; Lopez-Gonzales et al. 2011).

**Table 1:**
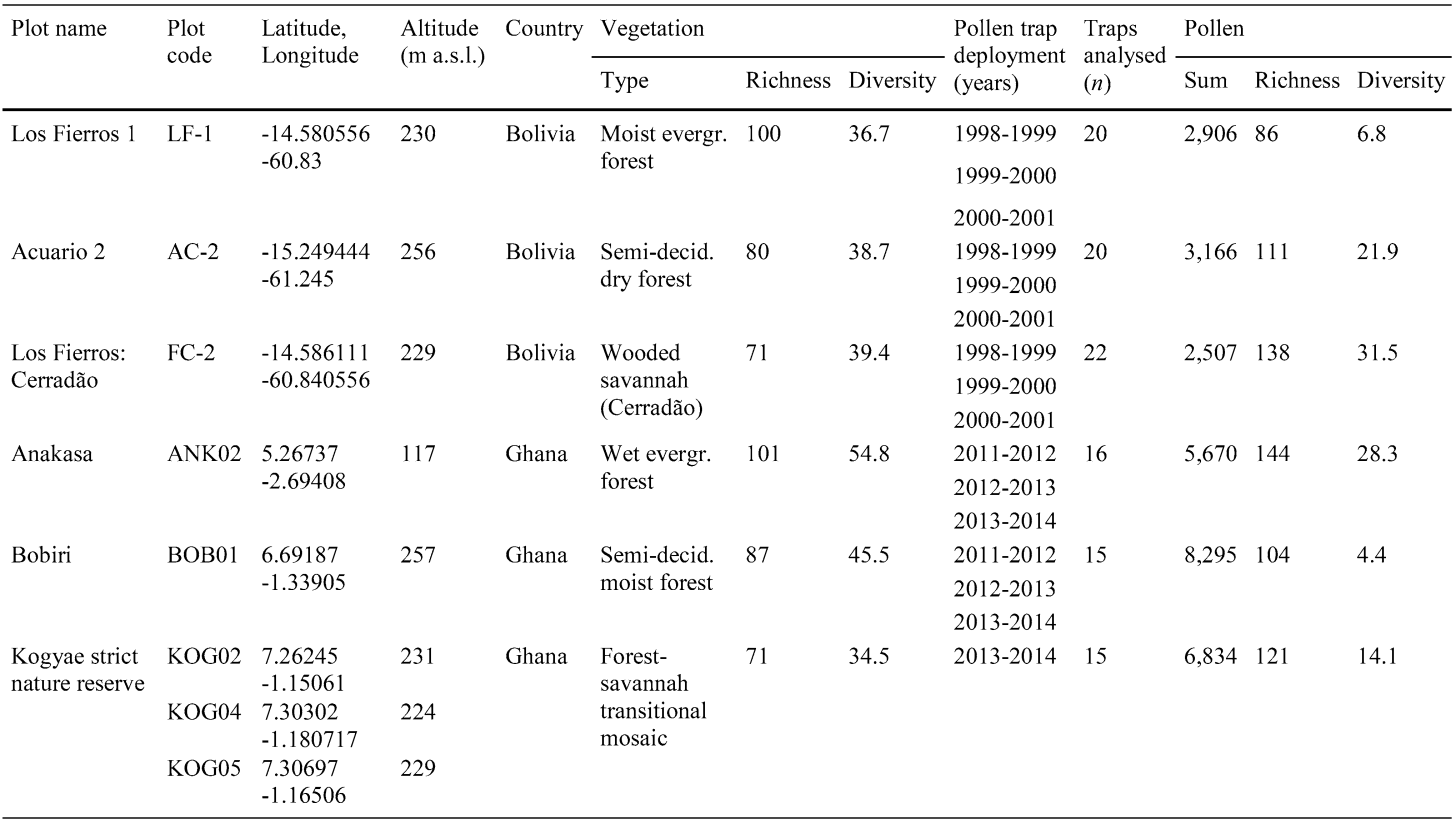
Vegetation study plot information from Bolivian (Neotropical) and Ghanaian (Palaeotropical) study regions. Data from Panfil (2001) and Lopez-Gonzalez et al. (2011)

**Fig. 1:**
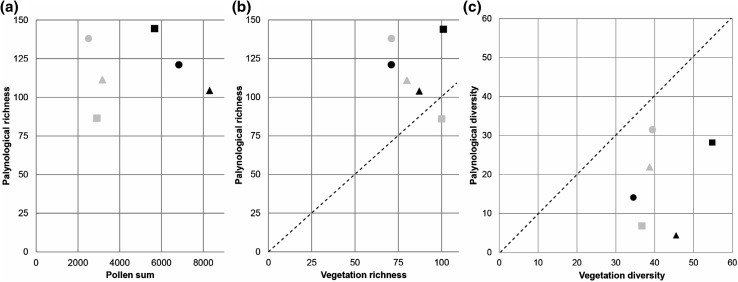
Plot scale relationships between: **a** palynological richness (Hill *N*_0_) and pollen sum (total number of pollen grains counted), **b** palynological richness and vegetation richness (both Hill *N*_0_), and **c** palynological diversity and vegetation diversity (both Hill *N*_1_). Grey symbols indicate data from Neotropical (Bolivia) plots: square = moist evergreen forest (LF1), triangle = semi-deciduous dry forest (AC2), and wooded savannah (FC2). Black symbols indicate data from Palaeotropical (Ghana) plots: square = wet evergreen forest (ANK02), triangle = semi-deciduous moist forest (BOB01) and circle = forest-savannah transition (KOG02, KOG04 and KOG05). Diagonal dashed line indicates 1:1 ratio of richness and diversity values

Pollen traps were processed using standard procedures (Moore et al. 1991) following the protocols in Gosling et al. (2003). Pollen grains were counted under 400 × and 1,000 × magnification using Nikon LABOPHOT (Bolivian traps, by WDG) and Nikon Eclipse 50i (Ghanaian traps, by ACMJ) microscopes. Pollen identifications were based on pollen reference collections (University of Leicester, The Open University, University of Amsterdam), pollen atlases and online resources (Bonnefille 1971; Bonefille and Rioellet 1980; Moore et al. 1991; Roubik and Moreno 1991; Colinvaux et al. 1999; Bush and Weng 2007; Vincens et al. 2007; Gosling et al. 2013). Target pollen sums (total number of pollen grains counted) for the Bolivian plots were > 100 grains per trap, which resulted in > 2,500 grains per vegetation study plot (Table 1; Fig. 1a). Target pollen sums for the Ghanaian traps were estimated using model 1 of the sample specific count method developed by Keen et al. (2014). Application of the Keen et al. (2014) model 1 resulted in pollen sums of > 5,500 grains per vegetation study plot (Table 1; Fig. 1a).

The number of distinct taxa (richness) within both pollen and vegetation data were calculated by summing the number of taxa identified in the respective assemblages for each plot; equivalent to Hill *N*_0_ (Hill 1973). To gain further insight into the diversity characteristics of the pollen and vegetation the Hill *N*_1_ diversity values for both were calculated (Hill 1973). The Hill *N*_1_ numbers were selected because they integrate both richness and evenness (how similar the abundance of different taxa within an assemblage are) aspects of the data (Hill 1973). The Hill *N*_1_ numbers (hereafter referred to as “diversity” for simplicity) of the pollen and vegetation assemblages for the plots were calculated using the Vegan package (Oksanen et al. 2017) for R (R Core Team 2015).

## Study sites

In the Neotropics (Bolivia) pollen traps from three vegetation study plots were examined: (1) Los Fierros 1 (LF-1); (2) Acuario 2 (AC-2); and (3) Los Fierros: Cerradão (FC-2). The plots all receive approximately 1,500 mm rainfall per year and experience a mean annual temperature of c. 25 °C. Analysis of the vegetation data revealed that these plots were representative of three distinct vegetation types: (1) moist evergreen rainforest (LF-1) characterised by large evergreen trees within the Vochysiaceae, Moraceae and Rubiaceae which formed a closed canopy; (2) semi-deciduous dry forest (AC-2) characterised by short woody species within the Fabaceae and Bignoniaceae; (3) Cerradão, or wooded savannah, (FC-2) characterised by woody species in the Guttiferae and Melastomataceae and grasses (Poacaeae) (Panfil 2001).

In the Palaeotropics (Ghana) pollen traps from five vegetation study plots were examined: (1) Ankasa (ANK02); (2) Bobiri (BOB01); (3) Kogyae Forest (KOG02); (4) Kogyae Transition (KOG04); (5) Kogyae Savannah (KOG05). The plots in the three regions (Ankasa, Bobiri and Kogyae) receive c. 2,000, c. 1,000 and c. 1,000 mm rainfall per year respectively, and all experience a mean annual temperature of c. 25 °C. Analysis of the vegetation data revealed that these plots were representative of three distinct vegetation types: (1) wet evergreen rainforest (ANK02) characterised by large trees in the Putranjivaraceae, Fabaceae and Combretaceae; (2) semi-deciduous moist forest (BOB01) characterised by woody taxa within the Malvaceae, Fabaceae and Cannabaceae; (3) forest-savannah transition (KOG02, KOG04 and KOG05) characterised by woody species in the Moraceae and Combretaceae, and grasses (Poaceae) (Lopez-Gonzales et al. 2011).

The vegetation from Bolivia and Ghana is broadly comparable because the study plots are spaced across the forest-savannah ecotone on the respective continents. It is important to note, however, that the Bolivian plots are relatively closer together (Bolivian plots all within c. 90 km, Ghanaian plots across c. 300 km). Consequently, the Bolivian traps are located within a landscape with a stronger mosaic (higher *beta* diversity) because they are all relatively closer to the forest-savannah transition (Killeen and Schulenberg 1998).

## Results

Despite the slight differences in the vegetation types the richness values for the evergreen forest, semi-deciduous forest and savannah plots in Bolivia and Ghana are remarkably similar (Table 1). Furthermore, the same trend in richness is observed between vegetation study plots on both continents, with the evergreen forest having the highest richness (101 Bolivia, 100 Ghana), semi-deciduous forest the intermediate (80 Bolivia, 87 Ghana) and the savannah the lowest (71 Bolivia, 71 Ghana) values. The richness of the modern pollen rain from the vegetation study plots varies over a similar magnitude to the vegetation richness (86–144), but does not co-vary with either pollen sum (Fig. 1a) or vegetation richness (Fig. 1b).

The diversity of the modern pollen rain from the Neotropical vegetation study plots increased from moist evergreen rainforest (6.8), through semi-deciduous dry forest (21.9), to wooded savannah (31.5) (Fig. 1c). The diversity of the Neotropical vegetation from the same plots is similar from all plots: moist evergreen rainforest = 36.7; semi-deciduous dry forest = 38.7; wooded savannah = 39.4.

The diversity of the modern pollen rain from the Palaeotropical vegetation study plots increases from the semi-deciduous moist forest (4.4), through the forest-savannah transition (14.1), to the wet evergreen rainforest (28.3) (Fig. 1c). The diversity of the Palaeotropical vegetation from the same plots shows a different trend, rising from forest-savannah transition (34.5), through semi-deciduous moist forest (45.5), up to wet evergreen forest (54.8).

## Discussion

### Tropical pollen and vegetation richness

The absence of any clear relationship between pollen richness and pollen sum suggests that the pollen rain has been sampled sufficiently to capture the bulk of the palynological richness (Fig. 1a). The absence of a clear relationship between the pollen and vegetation richness (Fig. 1b) suggests that, perhaps unsurprisingly, factors other than the plot (local) vegetation richness are playing an important role in determining palynological richness (Goring et al. 2013; Birks et al. 2016). Important factors likely to be influencing the pollen rain, and consequently the richness signal, are variations in flower structure and pollen dispersal mechanism (Bush 1995; Bush and Rivera 1998, 2001).

The absence of a straightforward relationship between palynological and vegetation richness suggests, that within the tropics, caution should be exercised when using this metric to interpret past vegetation richness levels. However, comparison of richness between high diversity (tropical) and low diversity (temperate) systems does seem to demonstrate that pollen richness does track vegetation diversity at a global scale. The range of palynological richness captured in the tropical traps presented here is 86–144 pollen types (Table 1), compared with richness values from temperate regions which are typically lower; for example 3–27 pollen types in > 500 modern lake samples in North America (Goring et al. 2013), and 9–45 pollen types in fossil records from a wide variety of temperate regions across the globe (Flenley 2005).

### Tropical pollen and vegetation diversity

There is a marked difference between the pattern of diversity recorded in the pollen and vegetation across the three vegetation types sampled from the Neotropics (Table 1). The similarity between the diversity of the vegetation likely reflects a low diversity of the moist evergreen forest plot relative to moist evergreen forest found elsewhere in the Neotropics (Gentry 1988). Two factors likely contribute to the low diversity of the vegetation in the Bolivian plots: (1) the young age of the moist evergreen forest (Mayle et al. 2000; Burbridge et al. 2004), these forests are just a few 1,000 years old and the high diversity levels typically associated with rainforest may not have had time to develop yet; and (2) the close proximity to the southerly extent of moist evergreen rainforest probably means some typical moist evergreen forest taxa are excluded due to drought stress.

The trend in palynological diversity across the three vegetation types probably reflects the high landscape (*beta*) diversity and the different vegetation structures (Killeen and Schulenberg 1998). The more open the vegetation structure in the plot is, the more likely regional (tens of kilometres) pollen rain from other vegetation types within the landscape is to contribute to the pollen rain collected within the pollen trap. Consequently, the wooded savannah records the highest palynological diversity because it is capturing pollen, not only from vegetation within the plot, but also from the vegetation mosaic landscape beyond. Conversely, the lowest palynological diversity is recorded in the moist evergreen forest, which has a dense closed canopy that results in the collection of a very local pollen signal dominated by a small number of wind pollinated taxa (Gosling et al. 2005).

The highest palynological and vegetation diversity in the three vegetation types sampled in the Palaeotropics is for the wet evergreen rainforest, but the lowest diversity values for the pollen are for the semi-deciduous moist forest, and the lowest diversity value for the vegetation is for the savannah (Table 1). The high diversity in the wet evergreen forest plot suggests the pollen is reflecting the high vegetation (*alpha*) diversity. The low diversity estimated for the semi-deciduous moist forest is due to the unevenness of the pollen data set caused by a high proportion of one taxon (specifically *Celtis* in the Cannabaceae; Julier 2017). The relatively elevated palynological diversity values for the forest-savannah transition vegetation likely reflect the pollen traps collecting material from a diverse landscape (high *beta* diversity), i.e. the forest-savannah data are derived from three different plots across a vegetation mosaic (Julier et al. 2017).

### Richness and diversity relationships and implications for interpreting the fossil record

**Fig. 2:**
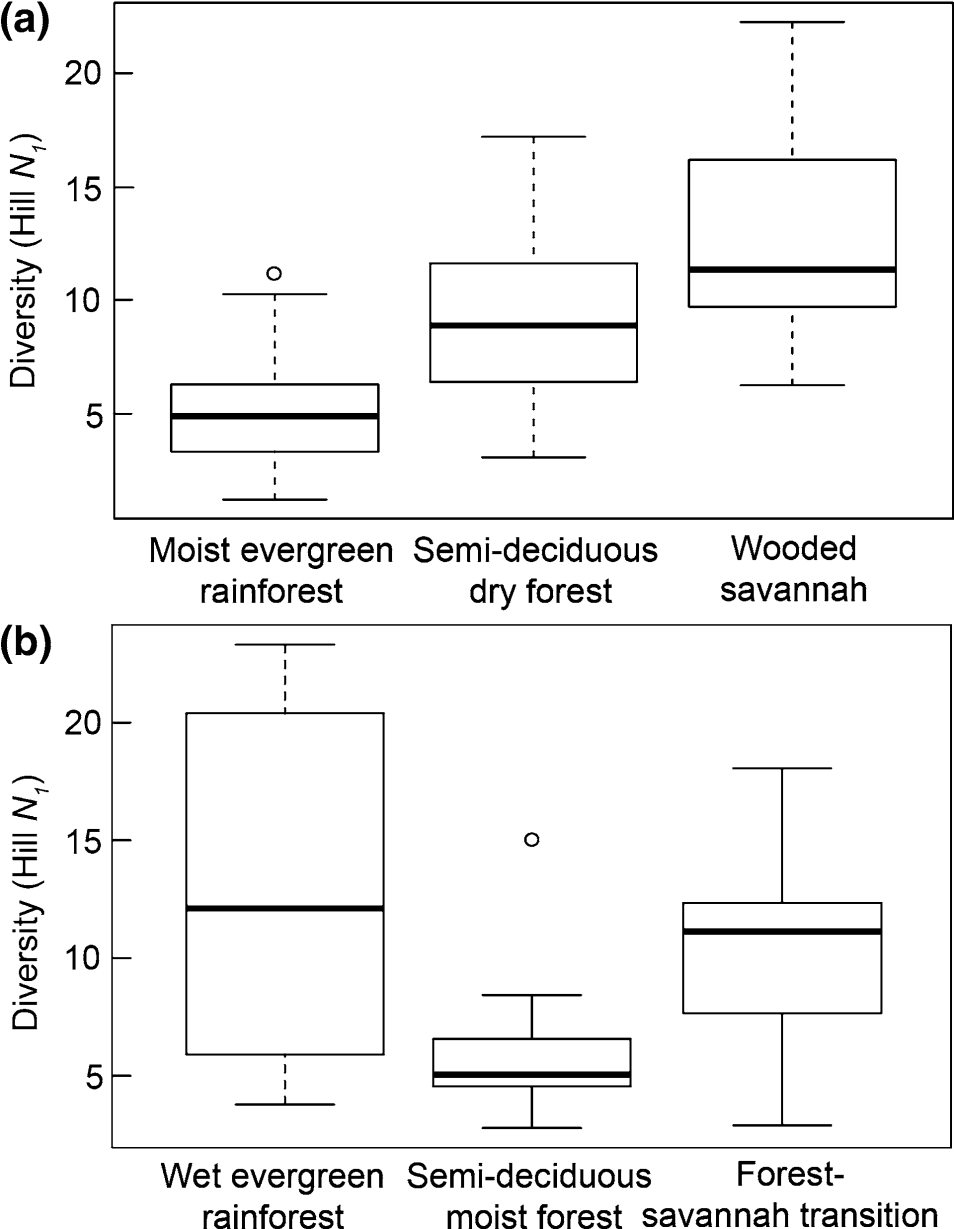
Palynological diversity (Hill *N*_1_) from moist evergreen forest, semi-deciduous forest, and savannah ecosystems in: **a** Neotropics (Bolivia), and **b** Palaeotropics (Ghana). Box and whisker plots show range of palynological diversity values obtained from individual pollen trap data. For full details of study plots see Table 1

The range of richness and diversity values from the Neotropical and Palaeotropical sites overall are broadly similar; plot scale palynological richness values in the Neotropics are 86, 111 and 138 *vs.* the Palaeotropics values of 144, 104 and 121 (Fig. 1), while median diversity values of traps in the Neotropics are 4.9, 8.9 and 11.4 *vs.* 12.1, 4.4 and 10.5 for the Palaeotropics (Fig. 2). Furthermore, the vegetation richness of the equivalent vegetation types across the continents differs by less than 10% and diversity of the two regions is comparable; 36.7, 38.7 and 39.4 in the Neotropics *vs.* 54.8, 45.5 and 34.5 in the Palaeotropics (Fig. 1c). The similarity of the richness and diversity values suggests that comparable factors are operating in both tropical regions to determine the way in which the vegetation richness/diversity is translated into palynological richness/diversity.

In the tropical vegetation study plots palynological richness values were found to be higher than those for the vegetation richness in all but one case (Fig. 1b), and lower terms of the diversity in all cases (Fig. 1c). The comparison of these two metrics, and different offset between the palynological and vegetation richness and diversity, provides insight into how the character of the pollen assemblage affects the diversity signal derived. It seems likely that, even when large pollen sums have been obtained to ensure that the pollen rain is well sampled (e.g. BOB01), inherent unevenness of pollen samples from the tropics can result in an underestimation of diversity relative to the vegetation. Unevenness of pollen samples in the tropics occurs due to high differentials in the under- and over-representation of taxa due to differences in flower structure and pollination strategy (Bush 1995; Gosling et al. 2009; Julier et al. 2017).

The relatively higher palynological richness values are probably due to a combination of: (1) the large source area sampled by the pollen traps, i.e. pollen from a highly diverse landscape is collected rather than just the pollen from the study plot, (2) the importance given to rare pollen types by this metric, i.e. tropical pollen counts often have many distinct “types” that occur at low numbers but are not assigned botanical affinities which inflate that richness calculation, and (3) the absence of plants < 10 cm dbh from the vegetation data potentially being represented in the pollen rain.

The reason for the lower diversity values is likely due to the poorer taxonomic resolution of the palynological data (typically genus and family) compared to the vegetation (usually species) (Bush and Rivera 2001), and the tendency for artificial pollen traps to be ‘swamped’ by one taxon, usually a wind pollinated plant, or one very close to that trap (Julier et al. 2017). The loss of diversity information in the fossil record demonstrates the need for the development of improved taxonomic tools if we are going to be able to use fossil pollen to provide an accurate window into past diversity change, e.g. Mander et al. (2013) and Julier et al. (2016).

Based on the modern pollen-vegetation data presented here it seems that in tropical ecosystems reconstructions of past vegetation diversity based on fossil pollen records are likely to be either: (1) a systematic over-representation if based on richness, or (2) an under-representation if based on a diversity metric that includes evenness. Consequently, the differential offset between metrics should be considered, and caution should be taken, when inferences of past vegetation diversity are being made.

The inability of the pollen to closely mirror the richness or diversity of the parent vegetation suggests that changes in pollen diversity observed in the fossil record should be treated with caution. The richness and diversity data from these tropical vegetation study plots suggest that the palynological diversity is determined by a combination of the diversity of the parent vegetation (*alpha* diversity), the pollination strategy of the taxa within that parent vegetation, and the source area from which the pollen rain is collected (*beta* diversity). Consequently, interpretation of past diversity trends from fossil pollen data, such as Jaramillo et al. (2006), should keep in mind the possible influence of shifts in pollination strategies through time, and control as much as possible for changes in source area, i.e. through the consideration of the sedimentary material from which the pollen was extracted.

## Conclusions

The palynological diversity estimated from pollen samples obtained through the deployment of artificial traps in forest and savannah vegetation in Bolivia (Neotropics) and Ghana (Palaeotropics) does not track the richness or diversity estimated from the vegetation in the study plots from which they were collected (Figs. 1, 2). The reason for the divergence of the palynological and vegetation richness and diversity is likely twofold: (1) there is variation in the dominant pollination strategy between different vegetation types, and (2) there is a difference in source area from which the pollen collected in the traps is derived related to the degree of canopy openness. There is a consistent off-set between richness (over-representation) and diversity (under-representation) estimates based on the pollen relative to the vegetation. Pollen richness values are higher than the vegetation probably due to the large number of rare (frequently unknown) pollen types that are often recorded in tropical pollen counts. Pollen diversity estimates are lower than vegetative diversity estimates regardless of the vegetation type suggesting that fossil pollen diversity under-estimates past vegetation diversity. Consequently new tools are required to improve the taxonomic resolution that can be extracted from the fossil record. Despite the differences between the metrics, and the consequent problems of relating palynological and vegetation richness and diversity within the tropics, it is, however, clear that on a global scale the high diversity of the tropical vegetation is reflected in the pollen rain it produces.
